# Potential Protective Activities of Extracts of *Phellinus linteus* and the Altered Expressions of *GSTM3* on Age-Related Cataract

**DOI:** 10.1155/2021/4313805

**Published:** 2021-01-21

**Authors:** Cheng-Hsiu Lin, Chun-Ching Shih

**Affiliations:** ^1^Department of Internal Medicine, Fengyuan Hospital, Ministry of Health and Welfare, Fengyuan District, Taichung City 42055, Taiwan; ^2^Graduate Institute of Biotechnology and Biomedical Engineering, College of Health Science, Central Taiwan University of Science and Technology, No. 666 Buzih Road, Beitun District, Taichung City 40601, Taiwan

## Abstract

Age-related cataract (ARC) is one of the leading causes of visual impairment and blindness worldwide among the elderly. Here, we used sodium selenite-induced cataract mouse model, which shares with similarities with human senile cataract to investigate whether the extracts of *Phellinus linteus* (PLE) could have the potential protective effects of ARC or not. The mice pups were randomly divided into 4 treatment groups (*n* = 7): (1) normal saline on postpartum day 26; (2) Na selenite injected s.c on day 26; (3) Na selenite s.c on day 26+ gavaged PLE (40 mg/kg) on days 26–47; and (4) Na selenite s.c on day 26 + resveratrol on days 26–47. On day 47, encapsulated lenses and plasma were analyzed for the levels of glutathione (GSH), superoxide dismutase (SOD), and malondialdehyde (MDA), a marker of lipid peroxidation. Lens epithelial cells (LECs) were also analyzed for the mRNA and protein expressions of glutathione S-transferase Mu (*GSTM3*). We demonstrated that PLE could prevent selenite-induced oxidative stress and cataract formation in mice by higher GSH and SOD and lower MDA in LECs, plasma, and liver tissues and the increases in the mRNA and protein expressions of *GSTM3* in LECs. Our data show the increasing oxidative stress in selenite-induced cataract mice. Our data reveal the benefits of PLE for preventive activity in selenite-induced cataract in mice and there is a good possibility that PLE could ameliorate human senile cataract.

## 1. Introduction

The increasing age problem tends to the one of major topics worldwide. Increasing age is considered to be the most important risk factor for cataract. Cataract is characterized by the opacification of eye lens. A considerable number of cataracts are classified as age-related cataract (ARC) [1]. ARC is one of phenomenon of ageing, as with an increase in age, the lens becomes more and more sclerotic and opaque.

The previous evidence shows that oxidant free radicals play a key role in mechanisms of incidence of ARC [2–4]. The pathogenesis of ARC is not completely understood, but oxidative stress with the accumulation of reactive oxygen species (ROS) in the lens is considered to be a major initiating factor in the formation of ARC [2–4]. Oxidative stress may result from an imbalance between the production of ROS and the cellular antioxidant defense mechanism [5]. Thus, supplementation with antioxidant nutrients is one reasonable approach to prevent cataract development.

**Figure 1:**
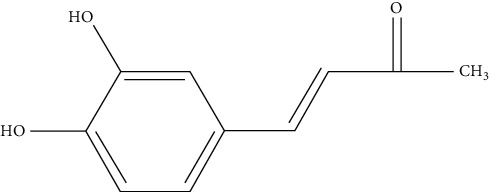
Chemical structure of 3,4-dihydroxybenzalactone.

*Phellinus linteus* has been used as foods and medicines in oriental countries. *P. linteus* consists of numerous bioactive compounds and is demonstrated to improve health and ameliorate various diseases [6]. Moreover, polyphenol is defined by the aromatic ring with more than two hydroxyl groups. Polyphenols could protect cell constituents against oxidative stress and reduce tissue damage by acting directly on reactive oxygen species (ROS) or by stimulating endogenous antioxidant defense systems [7]. 3, 4-Dihydroxybenzalactone (DAC) (Figure 1) is a component found only in fungi, especially in the *Phellinus* Genus [8]. 3, 4-Dihydroxybenzalactone is a polyphenol compound, and it has been shown to possess many activities, including antioxidant [9] and anti-inflammatory [10] as in demonstrated reports.

Cataracts consist of an opacification of the lens of the eye which impedes the passage of light and finally result in vision loss. Therefore, dietary components that possess antioxidant activity, such as polyphenols for instance, can be considered good candidates for human studies in the prevention and/or treatment such diseases. Among dietary components, the antioxidant capacity of certain polyphenols is well known, and these could be good candidates. A large number of preclinical studies support the involvement of resveratrol in clinical trials for the prevention and treatment of eye diseases induced by oxidative stress, such as age-related cataract [11]. Nevertheless, the protective potential activities of extracts of *Phellinus linteus* (PLE) on ARC remain still unknown in the animals.

The ability of selenite to cause cataracts was first reported by Ostadalova in 1978 [12]. Selenite administration by an injection to suckling rats leads to lipid peroxidation, hydrogen peroxide formation, and a decrease in reduced glutathione (GSH) levels in rat lenses [13]. Active oxygen generation has been demonstrated after reaction of selenite with reduced GSH [14]. Selenite cataract shows a number of general similarities to human senile cataract. In conclusion, selenium cataract is a useful *in vivo* rodent model for drug testing, despite some differences between human senile and selenium cataract.

Reactive oxygen species (ROS) decrease antioxidant levels in the lens and accelerate the damage to the lens epithelial cells (LECs), resulting in subsequent cataract development [15]. Glutathione (GSH) also functions as a coenzyme in glutathione S-transferases (GSTs) [16]. Glutathione S-transferases (GSTs) belong to an almost universal superfamily of enzymes that is present and has evolved in all aerobic organisms [16].

Glutathione S-transferase Mu 3 (*GSTM3*), which belongs to the *μ*-class subfamily, has a strong antioxidant function. Loss of functional *GSTM3* is involved in the pathogenesis of oxidative stress-related and age-related disease [17]. Glutathione S-transferase M3 is an antioxidative enzyme, so the dysfunction of *GSTM3* caused by promoter methylation may result in the dysfunction of the cellular antioxidant system and contribute to oxidative stress-associated LECs damage [18]. The downregulation of *GSTM3* in LECs not only reduced the viability but also made LECs more susceptible to oxidative stress, which would induce the formation of cataract [19].

In the present study, we will further investigate whether PLE prevents selenite cataract formation and examine its protective effects on the contents of GSH and malondialdehyde (MDA) (a maker of lipid peroxidation) in LECs, blood, and liver tissues. Based on one of the possible mechanisms, we also investigated whether PLE regulated expression of genes involved in the protective potential effect of ARC, and the mRNA and protein expressions of *GSTM3*, thus, contribute to the antioxidative stress-associated lens damage in selenite-induced ARC mice.

## 2. Materials and Methods

### 2.1. Chemicals

Antibodies to Glutathione S-Transferase Mu 3 (*GSTM3*) (no. CBS-PA009982LA01HU) were purchased from Cusabio Technology LLC (Cusabio, TX, USA). Secondary anti-rabbit antibodies were obtained from Jackson ImmunoRes. Lab., Inc. (West Grove, PA, USA).

### 2.2. Isolation and Determination of the Active Compound

The fruiting body of *Phellinus linteus* (1.5 kg, air dry weight) was powdered and extracted with 6 L of 95% ethanol at room temperature for three times. The extracts were filtered and combined together and then evaporated at 40°C (N–11, Eyela, Japan) to dryness under reduced pressure to give a dark brown residue (60 g). The yield obtained for 1 L is about 4%. The crude extract was suspended in H2O (1 L) and then partitioned with 1 L n-hexane (×2), 1 L Ethyl Acetate (EA) (×2), and 1 L n-butanol (×2), successively. It yielded five fractions. DBL was purified from EA soluble portion. A portion of the active EA fraction was subjected to silica gel chromatography using stepwise CHCl3-MeOH (9 : 1, 8 : 2, 1 : 1 *v*/*v*) as eluent. Final purification was achieved by preparative HPLC (Spherisorb ODS–2 RP 18, 5 *μ*m (Promochem), 250 × 25 mm, at flow rate of 10 *μ*L/min and UV detection at 375 nm). A fraction was recrystallized from ethylacetate to give 3,4-dihydroxybenzalacetone (DAC) (Figure 1). The procedure was as in a previously described report [6]. The spectral data of the isolated substance were yellow needles C10H10O3; 1 H NMR (DMSO, 400 MHz) *δ* 2.25 (s, 3H, CH3), 6.47 (d, 1H, *J* = 16 Hz, CH), 6.77 (d, 1H, *J* = 8.2 Hz, ArH), 7.05 (d, 1H, *J* = 2.0 Hz, ArH), 7.42 (d, 1H, *J* = 16 Hz, CH), 9.24 (s, 1H, OH), and 9.62 (s, 1H, OH); and 13C NMR (100 MHz, DMSO) *δ* 27.5, 115.1, 116.2, 122.1, 124.3, 126.2, 144.5, 146.0, 148.8, and 198.5.

### 2.3. Animals and Treatments

The animal protocol was approved by the guidelines of Central Taiwan University of Science and Technology, Taiwan, in accordance with the National Institutional Animal Care and Use Committee and approved by local animal ethics committee (Animal Ethics Committee, permit #109-CTUST-02) using a 3-week-old male BALB/cByJNarl obtained from the National Laboratory Animal Breeding Center. Animals were maintained under a 12 hr light:dark cycle and had free access to water and standard rodent chow. Twenty-eight BALB/cByJNarl male mice pups were divided into three experimental groups and one control group. In group 1 (*n* = 7), saline was gavaged on postpartum day 26. In group 2 (*n* = 7), sodium selenite (30 nmol/g bodyweight, Sigma Chemical Co., St. Louis, MO, USA) was injected subcutaneously on postpartum day 26 and gavaged with vehicle for 21 days. In group 3 (*n* = 7), the subcutaneous selenite injection on day 26 was gavaged with 21 daily doses of PLE (40 mg/kg body weight) on days 26–47. In group 4 (*n* = 7), the 30 nmol/g subcutaneous selenite injection on day 26 was gavaged with 21 daily doses of resveratrol (40 mg/kg, Sigma Chemical Co., St. Louis, MO, USA) on days 26–47. On day 47, encapsulated lenses and plasma were analyzed for levels of glutathione (GSH), superoxide dismutase (SOD), and malondialdehyde (MDA), a marker of lipid peroxidation. Lenses were also analyzed for the mRNA and protein expressions of *GSTM3* in the LECs.

### 2.4. MDA Analysis in LECs, Plasma, and Liver Tissues

The 10 mg liver tissues or lens epithelial cells were obtained to be homogenized on ice in 300 *μ*l of MDA Lysis Buffer (with 3 *μ*l BHT (100X) and then centrifuged (13,000 X *g*, 10 min.) to remove insoluble material. Lipid peroxidation of plasma, liver tissues, and lens epithelial cells was determined with a Lipid Peroxidation (MDA) Colorimetric/Fluorometric Assay Kit (BioVision; CA, U.S.A.) according to the manufacturer's instructions.

### 2.5. GSH Analysis in LECs, Plasma, and Liver Tissues

The 100 mg liver tissues or lens epithelial cells were obtained to be homogenized 0.5 ml 5% SSA and then centrifuged (8,000 X *g*, 10 min) to transfer the supernatant to a new tube and add ddH_2_O to reduce the concentration of SSA from 0.5 to 1%. GSH of plasma, liver tissues, or lens epithelial cells was determined with a GSSH/GSH Quantification Kit (Dojindo; MD, U.S.A.) according to the manufacturer's instructions.

### 2.6. SOD Analysis in LECs, Plasma, and Liver Tissues

The 100 mg liver tissues or lens epithelial cells were obtained, homogenized with 600 *μ*l sucrose buffer (0.25 M sucrose, 10 mM Tris, 1 mM EDTA, pH 7.4), and then centrifuged (10,000 X g for 60 minutes at 4°C) to transfer the supernatant to a new tube and add ddH_2_O to dilute the concentration of 20 X of plasma, 1000 X of liver, and 80 X lens epithelial cells determined with an SOD Assay Kit-WST (Dojindo; MD, U.S.A.) according to the manufacturer's instructions.

### 2.7. Relative Quantitation of mRNA Indicating Gene Expression

The experiment was determined using a procedure described elsewhere [20, 21]. Total RNA was reverse transcribed to cDNA in a reaction mixture containing buffer, dNTP, the oligo (dT) primer, and Moloney murine leukemia virus reverse transcriptase and then heated at 90°C for 5 min to terminate the reaction. The reverse transcription-polymerase chain reaction (RT-PCR) was performed in a final 25 *μ*l containing each forward (F) and reverse (R) primer, Tris-HCl containing Tween 20, and Taq DNA polymerase. Preliminary experiments were carried out with various cycles to determine the nonsaturating conditions of the PCR amplification for all the genes studied. The primers were as in Table 1. The products were run on 2% agarose gels and stained with ethidium bromide. The relative density of the bands was evaluated using AlphaEaseFc™ software. All the measured PCR products were normalized to the amount of cDNA of *β*-actin in each sample.

### 2.8. Western Blotting

The experiment was determined using a procedure described elsewhere [20, 21]. Protein extractions and immunoblots for the determination of targeted proteins including *GSTM3* were carried out on lenses from mice. Briefly, samples were homogenized with lysis buffer (pH 6.4) and protease inhibitors. Forty micrograms of each homogenate were used for SDS-PAGE and immunoblotting. Targeted *GSTM3* proteins were performed by the lenses of mice. Protein concentration was determined via BCA assay (Pierce). Equal amounts of protein were then diluted 4X in SDS sample buffer (62.5 mM Tris-HCl, 20% glycerol, 2% SDS, 75 M DTT, and 0.05% bromophenol blue) and subjected to SDS PAGE and were detected by western blotting with antibodies specific for targeted gene proteins. The density blotting was analyzed using Alpha Easy FC software (Alpha Innotech Corp., Randburg, South Africa).

### 2.9. Statistical Analysis

All of the results were presented as the mean and standard error. Whenever possible, data were subjected to analysis of variance and followed by Dunnett's multiple range tests, using SPSS software (SPSS Inc., Chicago, IL, USA). *p* < 0.05 was considered to be statistically significant.

## 3. Results

### 3.1. Body Weight and Absolute Tissue Weights

**Figure 2:**
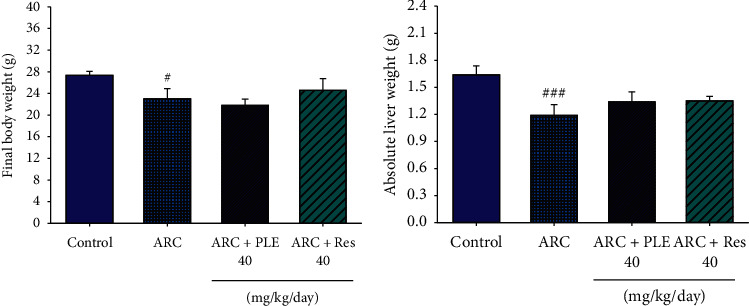
Effects of extracts of *Phellinus linteus* (PLE) on (a) final body weight and (b) absolute liver weights (g). *p*^#^ < 0.05, *p*^###^ < 0.001 compared with the control group; *p*^*∗*^ < 0.05, *p*^*∗∗∗*^ < 0.001 compared with the age-related cataract (ARC) + vehicle (distilled water) group. PLE, extracts of *Phellinus linteus*. Res, resveratrol.

As shown in Figure 2, at the end of the animal study, the final body weights of selenite-induced cataract (ARC) mice are significantly increased as compared with the control mice (*p* < 0.001). Following treatment with PLE or resveratrol, there is no significant effect in the final body weights as compared with the vehicle-treated ARC mice. At the end of the study, there is no statistical significance on the absolute liver weights between the vehicle-treated ARC group and the control group. Also, there is no statistical significance on absolute liver weights between PLE or resveratrol treatment and the vehicle-treated ARC group (Figures 2(a) and 2(b)).

### 3.2. The MDA Levels in LECs, Plasma, and Liver Tissues

**Figure 3:**
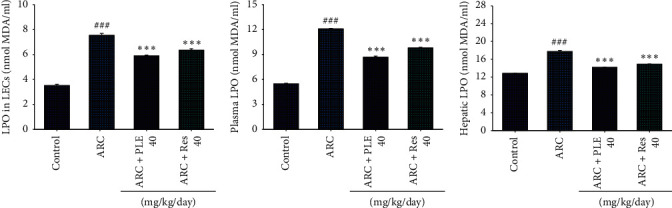
Effects of extracts of *Phellinus linteus* (PLE) on lipid peroxidation (LPO) contents in the (a) lens epithelial cell (LECs), (b) plasma, and (c) liver tissues of age-related cataract (ARC) mice. Malondialdehyde (MDA). *p*^###^ < 0.001 compared with the control group; *p*^*∗∗∗*^ < 0.001 compared with the age-related cataract (ARC) + vehicle (distilled water) group. PLE, extracts of *Phellinus linteus*. Res, resveratrol.

As shown in Figure 3, the MDA levels in the LECs, plasma, and liver tissue are significantly increased in the ARC group as compared to the control group (*p* < 0.001, *p* < 0.001, *p* < 0.001, respectively). Following treatment with PLE or resveratrol, the MDA levels in the LECs, plasma, and liver tissues are significantly decreased as compared with the vehicle-treated ARC group (Figures 3(a)–3(c)).

### 3.3. The GSH Levels in LECs, Plasma, and Liver Tissues

**Figure 4:**
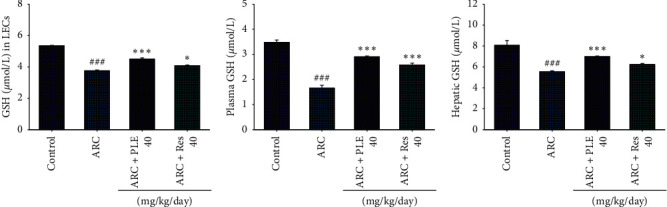
Effects of extracts of *Phellinus linteus* (PLE) on glutathione (GSH) in the (a) lens epithelial cell, (b) plasma, and (c) livers of age-related cataract (ARC) mice. *p*^###^ < 0.001 compared with the control group; *p*^*∗*^ < 0.05, *p*^*∗∗∗*^ < 0.001 compared with the age-related cataract (ARC) + vehicle (distilled water) group. PLE, extracts of *Phellinus linteus*. Res, resveratrol.

As shown in Figure 4, the GSH levels in the LECs, plasma, and liver tissues are significantly decreased in the ARC group as compared with the control group (*p* < 0.001, *p* < 0.001, *p* < 0.001, respectively). Following treatment with PLE, the GSH levels in the LECs and liver tissues are increased as compared with the vehicle-treated ARC group (*p* < 0.001). We found that PLE has greater effect than resveratrol on GSH levels in the LECs and liver tissue. Following treatment with PLE or resveratrol, the plasma GSH levels are significantly reduced as compared with the vehicle-treated ARC group (Figures 4(a)–4(c)).

### 3.4. The SOD Levels in LECs, Plasma, and Liver Tissues

**Figure 5:**
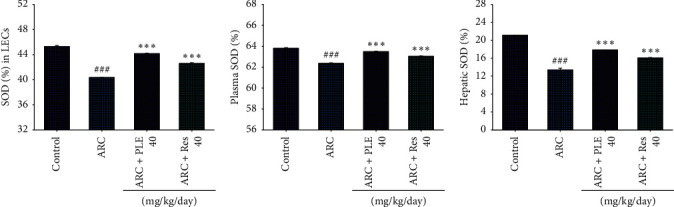
Effects of extracts of *Phellinus linteus* (PLE) on superoxide dismutase (SOD) in the (a) lens epithelial cells (LECs), (b) plasma, and (c) liver tissues of age-related cataract (ARC) mice. *p*^###^ < 0.001 compared with the control group; *p*^*∗∗∗*^ < 0.001 compared with the age-related cataract (ARC) + vehicle (distilled water) group. PLE, extracts of *Phellinus linteus*. Res, resveratrol.

As shown in Figure 5, the SOD levels in the LECs, plasma, or liver tissues are significantly decreased in the ARC group as compared with the control group (*p* < 0.001). Following treatment with PLE or resveratrol, the LECs, plasma, and hepatic SOD levels were increased as compared with the vehicle-treated ARC group (Figures 5(a)–5(c)).

### 3.5. The mRNA Levels of *GSTM3* in LECs

**Figure 6:**
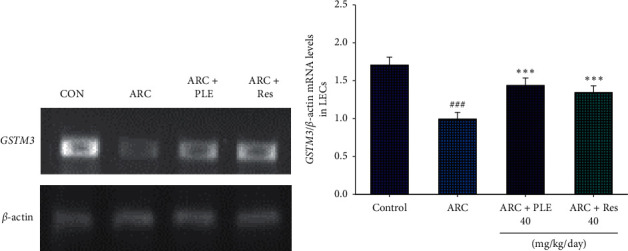
Semiquantative RT-PCR analysis on *GSTM3* mRNA levels in the LECs of the mice by oral gavage extracts of *Phellinus linteus* (PLE) (40 mg/kg body weight) or resveratrol (Res; 40 mg/kg body weight). (a) Representative image; (b) quantification of the ratio of target gene to *β*-actin mRNA expression. Total RNA (1 *μ*g) isolated from tissue was reverse transcripted by MMLV-RT; 10 *μ*L of RT products were used as templates for PCR. The levels of *GSTM3* mRNA were measured and quantified by image analysis. Values were normalized to *β*-actin mRNA expression. All values are means ± SE (*n* = 7). *p*^###^ < 0.001 compared with the control (CON) group; *p*^*∗∗∗*^ < 0.001 compared with the age-related cataract (ARC) + vehicle (distilled water) group.

As shown in Figure 6, the mRNA levels of *GSTM3* in LECs were lower in the ARC group than in the control (CON) group (*p* < 0.001). Administration of PLE or Res increased the mRNA levels of *GSTM3* in LECs as compared with the vehicle-treated ARC group (*p* < 0.001, *p* < 0.001, respectively) (Figures 6(a) and 6(b)).

### 3.6. The Protein Expressions of *GSTM3* in LECs

**Figure 7:**
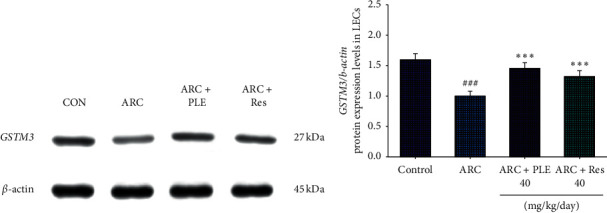
The protein expression levels of *GSTM3* in the LECs of selenite-induced age-related cataract (ARC) mice by oral gavage extracts of *Phellinus linteus* (PLE) (40 mg/kg body weight) or resveratrol (Res; 40 mg/kg body weight). (a) Representative image; (b) quantification of the *GSTM3* to *β*-actin. Protein was separated by 12% SDS-PAGE detected by western blot. All values are means ± SE (*n* = 7). *p*^###^ < 0.001 compared with the control group; *p*^*∗∗∗*^ < 0.001 compared with the age-related cataract (ARC) + vehicle (distilled water) group.

As shown in Figure 7, the protein expressions of *GSTM3* in LECs were lower in the ARC group than in the CON group (*p* < 0.001). Administration of PLE or Res significantly enhanced the protein expressions of *GSTM3* in LECs as compared with the vehicle-treated ARC group (*p* < 0.001, *p* < 0.001, respectively) (Figures 7(a) and 7(b)).

## 4. Discussion

The problem of increasing age worldwide is considered to be the most important risk factor for cataract with the opacification of lens. Age-related cataract (ARC) is one of the leading causes of visual impairment and blindness worldwide among the elderly. Although we could treat ARC by a surgery, nevertheless, it is associated with high cost and some risks [19]. Little is known about many health foods' act on ARC. *Phellinus linteus* contains polyphenol components, displays strong antioxidant activities, and promotes inhibition of lipid peroxidation, free-radical scavenging, and modulation of lipid and lipoprotein metabolism [6]. Nevertheless, the anti-ARC potential activities of PLE remain still unknown in sodium selenite-induced age-related cataract mice. Therefore, we hypothesized that the extracts of *Phellinus linteus* would effectively inhibit selenite-induced cataract formation in mice lenses.

In the present study, we investigate the potential protective effect and its underlying mechanism of extracts of *P. linteus* on ARC by selenite-induced cataract mouse model and compare with the positive antioxidant resveratrol. At the end of the animal experiment, lens, blood, and livers were collected for MDA, GSH, SOD, and *GSTM3* analysis.

Selenite administration results in lipid peroxidation, hydrogen peroxide formation, and a decrease in glutathione (GSH) levels in rodent lenses [13]. Our results showing the MDA levels in the LECs, plasma, and liver tissues are significantly increased in the ARC group as compared with the control were in agreement with other published studies [13]. Following treatment with PLE or resveratrol, the MDA levels in the LECs, plasma, and liver tissues are significantly decreased, implying that the oxidative stress and selenite-induced cataract is prevented by PLE acting as an antioxidant agent as caffeic acid phenethyl ester [22].

Selenite is believed to cause oxidation of cell components (including protein). Superoxide dismutase (SOD), GSH peroxidase, and catalase participate in the defense enzymes against oxidative stress induced by selenite [23]. GSH reductase and GSH S-transferase (GST) also function in this system [23, 24]. The increased H_2_O_2_ levels but the decreased lens NADPH and GSH contents further indicate that selenite probably functions as an oxidative agent [23, 25]. Previous evidence showed that selenite functions as an oxidative agent [23, 25]. In this study, our results showing that the GSH levels in the LECs, plasma, and liver tissues are significantly decreased in the ARC group as compared with the CON group are in agreement with other published selenite cataract studies [13, 14, 23–26]. Following treatment with PLE, the GSH levels in LECs, plasma, and liver tissues are increased. The crystalline lens contains a high concentration of GSH; this protects the lens from oxidant damage and toxic chemicals [27]. Human senile or experimental cataract formation is associated with progressive GSH decrease in the lens [28, 29]. Therefore, it can be suggested that PLE displays an increase in GSH levels by maintaining lens function and transparency by protecting sulfhydryl groups from oxidation [30, 31], implying that PLE acts an antioxidant by increasing GSH in selenite-induced age-related cataract mice.

The SOD levels in LECs, plasma, and liver tissues are significantly decreased in the ARC group as compared with the CON group. Following treatment with PLE or resveratrol, the SOD levels in LECs, plasma, and liver tissues were increased, implying that PLE displays an antioxidant role by increasing SOD with participation in the defense against oxidant stress [24] in selenite-induced age-related cataract mice.

Evidence has showed that mRNA and protein levels of *GSTM3* were significantly reduced in LECs and lens cortex of ARCs compared to the controls [19] and might be involved in the development of ARC. In this study, we found that the mRNA and protein levels of *GSTM3* were significantly reduced in LECs of ARCs compared to the controls, which are in agreement with the work Li et al. [19], and these results might be involved in the development of ARC. Administration of PLE or Res increased the mRNA and protein levels of *GSTM3* in LECs, implying that PLE or Res display an anti-ARC activity by *GSTM3* to prevent the age-related cataract by protecting the lens from oxidative stress, and an increased mRNA and expression level of *GSTM3* are observed in the LECs of selenite-induced age-related cataract treated with PLE or Res.

Our data reveal that PLE displays an anti-ARC role by a great antioxidant activity in MDA, GSH, and SOD levels in the LECs, plasma, and the liver tissues and the mRNA and protein levels of *GSTM3* in LECs. Also, we found that PLE's antioxidant activity is better than resveratrol in the LECs at the same dosages.

The increase of MDA levels in LECs, plasma, and liver tissues in the selenite group is indicative of the involvement of lipid peroxidation. Therefore, it seems plausible that PLE causes GSH level protection in addition to less lipid peroxidation with its potent antioxidant effect on LECs, plasma, and liver tissues.

**Figure 8:**
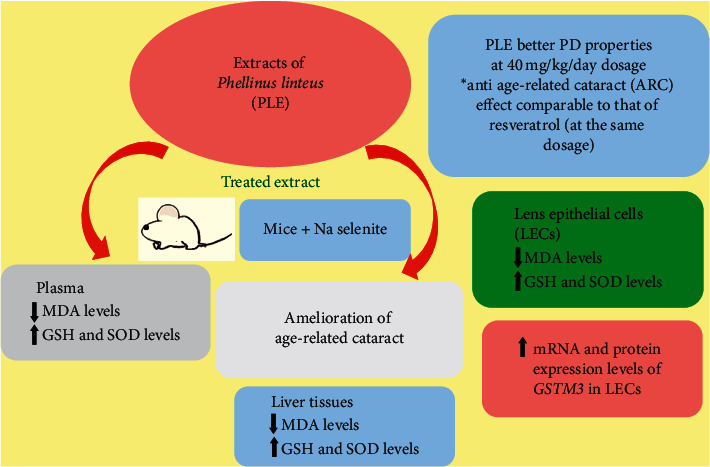
A proposed mechanism for PLE to ameliorate age-related cataract (ARC).

In conclusion, we investigated, for the first time, the preventive effect of PLE on selenite-induced cataract formation (Figure 8). These beneficial effects are associated with increased GSH, SOD, and *GSTM3*, but decreased MDA levels. These biochemical and molecular changes suggest an important role of oxidative stress in the selenite-induced cataractogenesis, with PLE displaying the role of an antioxidant.

## Figures and Tables

**Table 1:** Primers used in the present study.

Gene	Accession number	Forward primer and reverse primer	PCR product (bp)	Annealing temperature (°C)
GSTM3	NM_010359.2	F: GTCTGAGAAGACCACAGCAT R: TGTCCATAACTTGGTTCTCC	346	54

*β*-Actin	NM_007392	F: TCTCCACCTTCCAGCAGATGT R: GCTCAGTAACAGTCCGCCTAGA	99	55

## Data Availability

The data used to support the findings of this study are included with the main article. The fruiting body of *P. linteus* materials used in this study have been identified in the Department of Chinese Pharmaceutical Sciences and Chinese Medicine Resources, China Medical University where voucher specimens are deposited.

## Abbreviations

ARC: Age-related cataract
CON: Control
DAC: Dihydroxybenzalactone
GSH: Glutathione
GST: Glutathione S-transferase
GSTs: Glutathione S-transferases
*GSTM3*: Glutathione S-transferase Mu 3
LECs: Lens epithelial cells
LPO: Lipid peroxidation
MAD: Malondialdehyde
PLE: Extracts of *Phellinus linteus*
SOD: Superoxide dismutase
ROS: Reactive oxygen species
Res: Resveratrol
RT-PCR: Reverse transcription-polymerase chain reaction.
